# Profiling the small non-coding RNA transcriptome of the human placenta

**DOI:** 10.1038/s41597-021-00948-1

**Published:** 2021-07-02

**Authors:** Victor D. Martinez, David E. Cohn, Nikita Telkar, Brenda C. Minatel, Michelle E. Pewarchuk, Erin A. Marshall, E. Magda Price, Wendy P. Robinson, Wan L. Lam

**Affiliations:** 1 British Columbia Cancer Research Centre, 675 West 10th Ave, Vancouver, BC Canada; 2 IWK Health Centre, Halifax, NS Canada; 3 BC Children’s Hospital Research Institute, Vancouver, BC Canada; 4 Department of Medical Genetics, University of British Columbia, Vancouver, BC Canada

**Keywords:** RNA sequencing, Small RNAs

## Abstract

Proper functioning of the human placenta is critical for maternal and fetal health. While microRNAs (miRNAs) are known to impact placental gene expression, the effects of other small non-coding RNAs (sncRNAs) on the placental transcriptome are not well-established, and are emerging topics in the study of environmental influence on fetal development and reproductive health. Here, we assembled a cohort of 30 placental chorionic villi samples of varying gestational ages (*M* ± SD = 23.7 ± 11.3 weeks) to delineate the human placental sncRNA transcriptome through small RNA sequence analysis. We observed expression of 1544 sncRNAs, which include 48 miRNAs previously unannotated in humans. Additionally, 18,003 miRNA variants (isomiRs) were identified from the 654 observed miRNA species. This characterization of the term and pre-term placental sncRNA transcriptomes provides data fundamental to future investigations of their regulatory functions in the human placenta, and the baseline expression pattern needed for identifying changes in response to environmental factors, or under disease conditions.

## Abstract (summary)

**Table:** 

Measurement(s)	small non-coding RNA
Technology Type(s)	small RNA sequencing assay
Sample Characteristic - Organism	Homo sapiens

Machine-accessible metadata file describing the reported data: 10.6084/m9.figshare.14233229

## Background & Summary

The placenta is essential for the maintenance of pregnancy and the regulation of fetal growth and development^1^. Regulation of gene expression through epigenetic modifications^2^ and transcription factor availability^3^ is critical for healthy placental functioning^4–6^.

Small non-coding RNAs (sncRNAs) have the ability to regulate gene expression through a variety of epigenetic and post-transcriptional mechanisms^7^. Certain sncRNA subtypes, including microRNAs (miRNAs), have been associated with gene deregulation in pregnancy-associated diseases, including preeclampsia^8^. Specifically, miRNAs originating from the chromosome 14 and 19 miRNA clusters (C14MC and C19MC, respectively) have established contributions to placental gene regulation^9–11^.

However, studies of placental sncRNAs have focused almost exclusively on canonical miRNAs^12,13^, and not on other RNA species, such as PIWI-interacting RNAs (piRNAs), small nucleolar RNAs (snoRNAs), and miRNA variants (isomiRs) that can also influence the genetic and epigenetic regulation of transcription^14–16^. Furthermore, past efforts to identify human miRNAs have typically prioritized those that are highly expressed across multiple tissues^17^. MiRNA discovery efforts that focused on individual tissues have successfully identified novel, tissue-specific miRNAs that had previously been overlooked^18,19^, but the placenta has yet to be studied in this fashion. As a resource for future placental biology investigations, we have profiled and quantified the expression of annotated sncRNAs and determined the expression pattern of novel (previously-unannotated) miRNAs within the human placenta.

We isolated and sequenced the small RNA fractions of 32 placental (chorionic villi) samples of varying fetal sexes and gestational ages, 30 of which met our threshold for total high-quality reads (Table 1). Trimmed sequencing reads were input into the miRMaster platform, which performs quality filtering, aligns reads to annotated sncRNAs, quantifies sncRNA expression, and predicts novel miRNA sequences^20^. We considered a sncRNA to be ‘placentally expressed’ if it was present at ≥ 1 read per million (RPM) in at least 10% (3/30) of the placental samples. We considered a sncRNA to be expressed during a given trimester if it was present at ≥ 1 RPM in at least 10% of samples from that trimester. Raw data are made available for investigating sncRNA expression related to specific biological features^21^.

**Table 1:** Clinical data for analyzed placental samples.

Sample	Observed NTD*	GA^†^ (weeks)	Trimester	Sex	Processing Time (hours)	RQS^‡^
MX1355-C6RGTANXX-2-AAGCTA	No	11	1	Female	1	4.88
MX1355-C6RGTANXX-2-ACATCG	No	7	1	Female	2	5.11
MX1355-C6RGTANXX-2-CAAGTT	No	7	1	Female	1.5	6.32
MX1356-C6RGTANXX-3-CGGCCT	No	10	1	Male	1.33	5.08
MX1356-C6RGTANXX-3-TAGTTG	No	10	1	Male	2	6.24
MX1304-C5JC4ACXX-4-CCGGTG	Yes	22	2	Female	24	4
MX1305-C5JC4ACXX-5-TGTTGG	Yes	20	2	Male	48	3.6
MX1306-C5JC4ACXX-6-GTATAG	Yes	23	2	Female	48	3.4
MX1307-C5JC4ACXX-7-AGCATC	Yes	22	2	Male	24	4
MX1310-C5JC1ACXX-4-GGAACT	Yes	19	2	Female	24	4
MX1310-C5JC1ACXX-4-TGACAT	Yes	22	2	Male	96	3.4
MX1303-C5JC4ACXX-3-TAGGAT	No	17	2	Male	72	3.3
MX1307-C5JC4ACXX-7-CAGGCC	No	18	2	Female	24	4
MX1310-C5JC1ACXX-4-CTCTAC	No	24	2	Female	192	2.6
MX1310-C5JC1ACXX-4-GGACGG	No	19	2	Male	144	3.5
MX1355-C6RGTANXX-2-CATTCA	No	15	2	Female	168	NA
MX1355-C6RGTANXX-2-GGAACT	No	16	2	Female	168	3.11
MX1356-C6RGTANXX-3-CCTTGC	No	19	2	Female	120	3.22
MX1356-C6RGTANXX-3-GCGTGG	No	14	2	Female	72	2.97
MX1356-C6RGTANXX-3-GTATAG	No	19	2	Female	24	3.44
MX1357-C6RGTANXX-4-TCTGAG	No	22	2	Male	96	3.54
MX1356-C6RGTANXX-3-ATGGCA	No	40	3	Male	48	2.83
MX1356-C6RGTANXX-3-GCTGTA	No	40	3	Female	48	2.58
MX1356-C6RGTANXX-3-TGACAT	No	39	3	Male	72	2.98
MX1357-C6RGTANXX-4-AATTAT	No	38	3	Female	24	3.27
MX1357-C6RGTANXX-4-AGTCTT	No	40	3	Female	1.66	2.87
MX1357-C6RGTANXX-4-CATGGG	No	39	3	Male	15.5	2.78
MX1357-C6RGTANXX-4-GCCTAA	No	40	3	Male	24	3.88
MX1357-C6RGTANXX-4-GTAGCC	No	39	3	Male	1	3.58
MX1357-C6RGTANXX-4-TATCGT	No	39	3	Female	48	2.99

*NTD: neural tube defect. ^**†**^GA: gestational age. ^‡^RQS: RNA quality score.

**Fig. 1:**
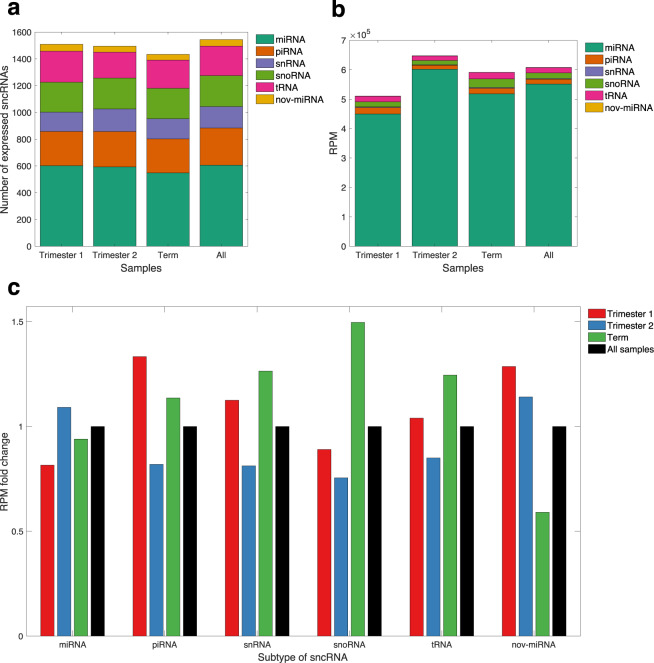
Summary of the quantity and expression levels of placentally expressed sncRNAs. **(a)** Count of sncRNAs, divided by subtype, that meet the expression cutoff (≥ 1 RPM in ≥ 10% of samples), across the entire sample cohort (n = 30), as well as across samples from a particular trimester. nov-miRNA: novel miRNA. **(b)** Mean total expression of all placentally expressed sncRNAs, divided by subtype, by trimester. **(c)** Mean total expression of all placentally expressed sncRNAs in each trimester, divided by subtype, and normalized relative to the mean total expression of sncRNAs of that subtype across all samples.

A total of 1544 distinct sncRNAs were placentally expressed, 81% of which met our expression threshold across all trimesters (Fig. 1a)^21^. Due to this similarity, all subsequent characterization considers only these 1544 placentally expressed sncRNAs, which include miRNAs, piRNAs, snoRNAs, small nuclear RNAs (snRNAs), and transfer RNAs (tRNAs).

A total of 654 miRNAs were placentally expressed, along with 277 piRNAs, 231 snoRNAs, 161 snRNAs, and 221 tRNAs (Fig. 1a). For all trimesters, miRNA reads made up a large majority (88–93%) of the total sncRNA reads (Fig. 1b). Of the 654 identified miRNAs, 48 were novel miRNA sequences (Online-only Table 1, GSE164178)^21^. In both individual features, such as length and GC content, and composite metrics, such as novoMiRank score^22^ and miRMaster-computed probability of their precursor being a true precursor, these novel miRNAs closely resemble the annotated placentally expressed miRNAs (Table 2).

**Table 2:** Comparison between annotated and novel placentally expressed miRNAs.

MiRNA Type	Length (nt)	GC Content (%)	NovoMiRank Score	MiRMaster-Assigned Probability
Annotated*	21.8 ± 1.2	49.0 ± 11.9	0.87 ± 0.21	0.83 ± 0.10
Novel^†^	22.0 ± 0.7	46.9 ± 8.7	0.92 ± 0.15	0.74 ± 0.06

All values are given as the mean, plus or minus the standard deviation. *The novoMiRank score and miRMaster-assigned probability values for annotated placentally expressed miRNAs were calculated from the annotated miRNAs that miRMaster also predicts^20^ to be true miRNAs (540 out of 606). ^†^The novoMiRank score and miRMaster-assigned probability values for novel placentally expressed miRNAs were calculated from all (48) novel miRNAs predicted by miRMaster.

In addition, there were 18,003 distinct isomiRs, which are natural variations of canonical miRNAs^23^, that were placentally expressed^21^. Additions or deletions of nucleotides at the 3′ end of isomiRs were far more prevalent (72% of all placentally expressed isomiRs) than at the 5′ end (18%) (Table 3). The relative proportions of isomiR types were similar across trimesters, but a greater total number of isomiRs were expressed during Trimesters 1 and 2 (17,870 and 16,541, respectively), than in term samples (12,444) (Table 3).

**Table 3:** Count of placentally expressed isomiRs by modifications and trimester.

	IsomiR Modification								
	5′ addition	5′ deletion	No 5′ change	3′ addition	3′ deletion	No 3′ change	No substitutions	1–2 substitutions	Total
**Trimester 1**	1080 (6%)	1927 (11%)	14,863 (83%)	5721 (32%)	7264 (41%)	4885 (27%)	2356 (13%)	15,514 (87%)	17,870
**Trimester 2**	1034 (6%)	2033 (12%)	13,474 (81%)	4362 (26%)	7380 (45%)	4799 (29%)	2342 (14%)	14,199 (86%)	16,541
**Term**	777 (6%)	1718 (14%)	9949 (80%)	4253 (34%)	4758 (38%)	3433 (28%)	2258 (18%)	10,186 (82%)	12,444
**All**	1074 (6%)	2161 (12%)	14,768 (82%)	5308 (29%)	7624 (42%)	5071 (28%)	2470 (14%)	15,533 (86%)	18,003

For each sncRNA subtype, we calculated the fold change in RPM values for each trimester relative to the average RPM for all samples. Expression of miRNAs peaks in second trimester samples, while expression of piRNAs, snRNAs, snoRNAs, and tRNAs is lowest in second trimester samples (Fig. 1c). PiRNA expression is highest in first trimester samples, while snRNA, snoRNA, and tRNA expression is highest in term samples (Fig. 1c).

**Fig. 2:**
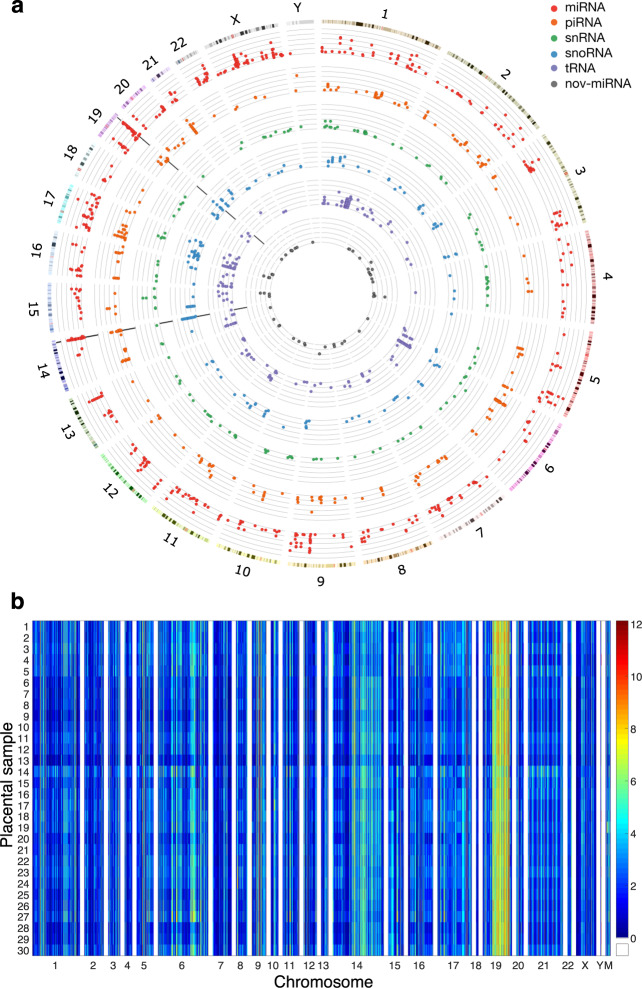
Illustration of the genomic locations of placentally expressed sncRNAs. **(a)** Circos plot ^29^ depicting the genomic location and mean log(1+x)-scaled expression level of all placentally expressed sncRNAs, including novel miRNAs. SncRNAs expressed from multiple genomic loci are shown at all such loci. Radial black lines within the chromosome 14 and 19 sectors indicate the positions of the C14MC and C19MC sncRNA clusters, respectively. **(b)** Heatmap displaying the log(1+x)-scaled RPM expression values of all placentally expressed known sncRNAs, divided by sample. SncRNAs expressed from multiple genomic loci are shown at all such loci. Samples are numbered in identical order to Table 1. M: mitochondrial chromosome.

SncRNAs of all subtypes, including novel miRNAs, were found to be expressed from almost all chromosomes (Fig. 2a). Regions of high miRNA expression were found on chromosomes 14 and 19, at the well-characterized C14MC and C19MC clusters (Fig. 2b).

## Methods

### Sample acquisition

This study used 32 de-identified chorionic villi samples collected at the BC Women’s Hospital and Health Centre from first trimester, second trimester, and term pregnancies, including 14 that had been previously collected^24^. Thirty of the samples had sufficient (> 1 million) high-quality sequencing reads to be included in further analysis. First trimester samples were obtained from elective terminations, while second trimester terminations were due to various fetal demise conditions, including but not limited to anencephaly, spina bifida, and preterm membrane rupture. Mode of delivery for 3/9 term samples was caesarean section. Cases with known chromosome abnormalities were excluded. Six of the samples were from fetuses that were phenotypically classified as having neural tube defects (Table 1).

For all cases ascertained before the termination of pregnancy, written consent was obtained. For all cases obtained retrospectively from pathological autopsy specimens, biospecimens were de-identified and all links to clinical data were removed. No identifiable information for any cases is presented in this publication. Ethics approval was obtained from the joint University of British Columbia/Children’s Hospital and Women’s Health Centre of British Columbia Research Ethics Board (H10-01028, H16-02280, and H04-70488).

After placental membrane removal, 30 mg of chorionic villi was sampled from the fetal-facing side of the placenta. Processing time after delivery ranged from 1–192 hours, and samples were RNAlater preserved. During the time of extraction, excess RNAlater was removed by blotting with Kimwipes (Kimberly-Clark, USA), after which samples were homogenized in the Next Advance Bullet Blender Tissue Homogenizer (Next Advance, USA), using the 3.2 mm Stainless Steel Beads (Next Advance, USA), with 1 ml of TRIzol reagent (ThermoFisher Scientific, USA). Samples were then incubated for 5 minutes at room temperature (RT), and then centrifuged at 7000 rpm for three minutes. 200 ml of chloroform (ThermoFisher Scientific, USA) was added to the supernatant, which was thoroughly mixed by inversion, and incubated for five minutes at RT. Samples were centrifuged at 4 °C at 9000 rpm for 20 minutes, and 500 μl of isopropanol (ThermoFisher Scientific, USA) was added to the aqueous phase, which was mixed by inversion and incubated for 10 minutes at RT. Samples were further centrifuged at 4 °C at 9000 rpm for 15 minutes. Supernatant was discarded and RNA pellet was washed in 75% ethanol (Commercial Alcohols, diluted with Ultrapure Distilled Water-RNAse/DNAse free, Gibco-LifeTechnologies) by gently inverting. Samples were centrifuged at 4 °C at 9000 rpm for 10 minutes. Supernatant was discarded, and RNA pellet was air-dried for five minutes at RT. RNA was eluted in 50 μl of nuclease-free water (Ultrapure Distilled Water-RNAse/DNAse free, Gibco-LifeTechnologies). Genomic DNA removal was carried out using the RNase-Free DNase Set (Qiagen, Germany). RNA concentration was measured on a Nanodrop 2000 (ThermoFisher Scientific, USA). RNA quality was assayed on an Agilent Bioanalyzer 2100 (Agilent, USA). Prior to sequencing, small RNA fractions were depleted of ribosomal RNA by hybridization, using the NEBNext rRNA Depletion Kit (New England BioLabs, USA).

### Sequencing and quality control

Samples were sequenced at Canada’s Michael Smith Genome Sciences Centre in Vancouver, using their standard ribodepleted strand-specific RNA (ssRNA) sequencing protocol^25^. This protocol includes plate-based ssRNA library construction, followed by sequencing on an Illumina HiSeq 2500 using the 3′ TruSeq small RNA adapter. No negative sequencing controls or positive spike-in controls were used.

Sequencing reads were subjected to a series of quality control steps, in order to trim adapters and discard reads that were < 16 nucleotides. Trimmed reads (FASTQ) were processed through the miRMaster platform (accessed on Mar. 2018), under default settings^20^. Reads with a Phred quality score < 20 were discarded, and samples with < 1 million remaining reads were excluded from further analysis (2/32 samples).

### Detection of annotated sncRNAs

MiRMaster maps reads to the human genome (hg38) using Bowtie 2 and assigns them to different classes of sncRNAs (miRNA, piRNA, snRNA, snoRNA, or tRNA). Reads that nearly mapped to annotated miRNAs (miRBase v21), with 5′ or 3′ additions or deletions of nucleotides and up to two mismatches, were classified as isomiRs. Reads were then quantified by miRMaster and scaled on a per sample basis by units of reads per million. For sncRNA sequences that mapped to multiple locations in the genome, only the reads derived from the locus with the highest mean expression were retained. Sequences expressed at ≥ 1 RPM in ≥ 10% (3/30) of the samples were considered to be ‘placentally expressed’.

### Discovery of novel (previously-unannotated) miRNAs

All reads not aligning with annotated sncRNAs were assessed by miRMaster, using a machine learning algorithm trained to classify sequences as true or false miRNA precursors. MiRMaster employs the AdaBoost algorithm, trained on a set of 216 miRNA features, including nucleotide ratios, free energy metrics, and folding metrics^20^. Prospective miRNA precursors were also scored by novoMiRank. These scores represent the extent to which a prospective precursor differs from early miRBase-catalogued precursors in 24 features, including nucleotide composition, loop length, and the genomic proximity of other miRNA precursors^22^. Prospective miRNA precursors were filtered according to their miRMaster-assigned probability of being a true precursor (≥ 65%) and their novoMiRank score (≤ 1.5), and the corresponding prospective miRNAs were filtered by their expression level (≥ 1 RPM in ≥ 10% of samples). For novel miRNA sequences that could be derived from multiple prospective miRNA precursors, only the sequence derived from the prospective precursor with the highest miRMaster-assigned probability of being a true precursor was retained.

## Data Records

FASTQ files containing raw sequencing reads can be accessed through the *NCBI Sequence Read Archive*^26^. CSV files detailing the reads per million expression values of sncRNAs in each placental sample can be accessed through the *Gene Expression Omnibus*^21^. Data are provided for all sncRNAs and isomiRs with at least one read in one sample, not only those that were placentally expressed. Similarly, data are provided for all candidate novel miRNAs, including those that did not pass filters for expression, novoMiRank score, or miRMaster-assigned probability of being a true precursor. Each sample has a separate file for expression of annotated sncRNAs, novel miRNAs, and isomiRs.

## Technical Validation

RNA quality score (RQS) was found to correlate with both sample processing time (Spearman’s ρ = −0.55, *p* = 1.9 × 10^−3^) and gestational age (Spearman’s ρ = −0.64, *p* = 1.9 × 10^−4^) (Table 1).

**Fig. 3:**
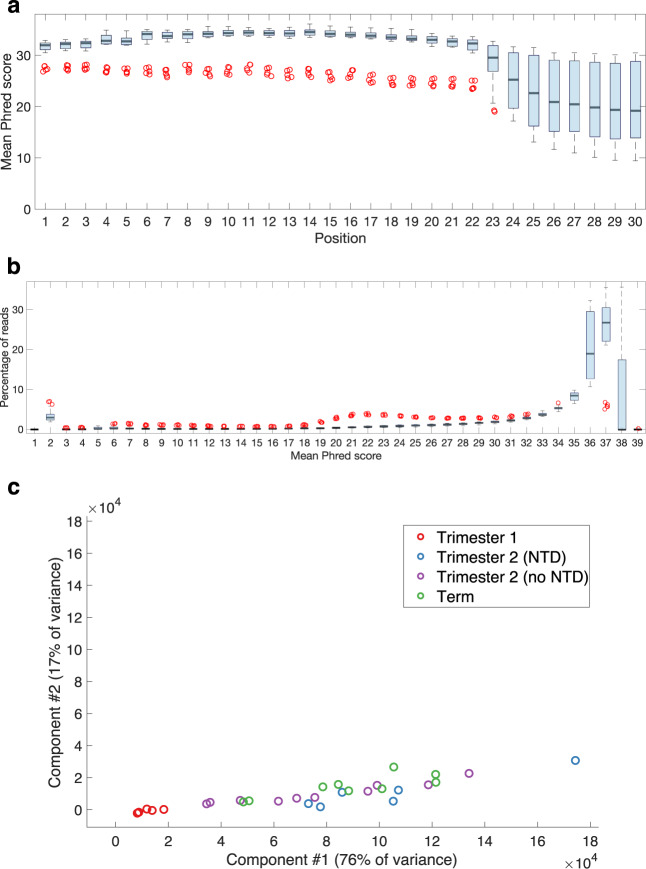
Summary of sequencing quality metrics for all analyzed placental samples (n = 30). **(a)** Boxplot of the mean Phred scores for each sample at each position of a sequencing read. **(b)** Boxplot of the percentage of reads within each sample that have a given mean Phred score. **(c)** Plot of all placental samples with respect to the first two principal components derived from the expression levels of all placentally expressed sncRNAs. NTD: neural tube defect.

In order to ensure the accuracy of sequencing reads, reads with Phred scores < 20 were discarded. Prior to this filtering, FastQC v0.11.9 was used to assess the overall sequencing quality for each sample^27^. The mean (SD) pre-filtering Phred score for individual samples was 31.83 ± 2.99 (Table 4). Samples with the library ID ‘MX1355’ (n = 5) possessed a lower mean (SD) Phred score of 25.37 ± 0.67, which most likely represents a batch effect (Table 4). The median sample yielded an average Phred score of > 20 at positions 1–27 of the trimmed reads, indicating that mature miRNAs (length 18–25 nt) were being accurately quantified (Fig. 3a). Reads with average Phred scores of 35–37 are the most abundant in all samples (Fig. 3b). The mean (SD) GC content for individual samples was 48.7 ± 1.5% (Table 4).

**Table 4:** Sequencing quality metrics for placental samples.

Sample	Total Reads	GC Content (%)	Mean Phred Score	Mean Read Length (nt)
MX1355-C6RGTANXX-2-AAGCTA	4,540,838	49	26.0	25.6
MX1355-C6RGTANXX-2-ACATCG	49,777,939	49	26.1	25.0
MX1355-C6RGTANXX-2-CAAGTT	10,485,967	50	24.6	26.1
MX1356-C6RGTANXX-3-CGGCCT	9,114,012	49	32.5	26.6
MX1356-C6RGTANXX-3-TAGTTG	3,898,000	51	32.9	25.4
MX1304-C5JC4ACXX-4-CCGGTG	18,543,258	47	32.4	22.3
MX1305-C5JC4ACXX-5-TGTTGG	32,804,195	47	33.7	22.1
MX1306-C5JC4ACXX-6-GTATAG	21,833,816	48	34.2	21.9
MX1307-C5JC4ACXX-7-AGCATC	29,085,135	47	33.7	22.2
MX1310-C5JC1ACXX-4-GGAACT	48,656,886	48	33.0	22.6
MX1310-C5JC1ACXX-4-TGACAT	41,920,665	50	32.7	22.5
MX1303-C5JC4ACXX-3-TAGGAT	13,492,427	48	32.7	22.4
MX1307-C5JC4ACXX-7-CAGGCC	17,782,226	48	33.2	22.3
MX1310-C5JC1ACXX-4-CTCTAC	26,491,193	55	32.3	22.3
MX1310-C5JC1ACXX-4-GGACGG	46,481,796	49	33.1	22.5
MX1355-C6RGTANXX-2-CATTCA	17,081,543	49	24.9	25.4
MX1355-C6RGTANXX-2-GGAACT	20,574,222	49	25.3	25.3
MX1356-C6RGTANXX-3-CCTTGC	17,471,028	49	32.9	24.5
MX1356-C6RGTANXX-3-GCGTGG	17,252,529	49	32.4	24.4
MX1356-C6RGTANXX-3-GTATAG	32,558,877	48	33.2	23.6
MX1357-C6RGTANXX-4-TCTGAG	38,455,096	49	32.8	23.8
MX1356-C6RGTANXX-3-ATGGCA	7,753,137	48	33.3	24.1
MX1356-C6RGTANXX-3-GCTGTA	38,298,019	49	33.0	24.2
MX1356-C6RGTANXX-3-TGACAT	52,029,262	48	32.8	23.3
MX1357-C6RGTANXX-4-AATTAT	5,643,655	47	33.7	23.5
MX1357-C6RGTANXX-4-AGTCTT	38,435,863	48	33.8	23.6
MX1357-C6RGTANXX-4-CATGGG	9,525,262	49	33.5	24.2
MX1357-C6RGTANXX-4-GCCTAA	31,394,752	48	33.4	24.1
MX1357-C6RGTANXX-4-GTAGCC	19,268,464	48	33.7	24.1
MX1357-C6RGTANXX-4-TATCGT	42,216,891	48	33.4	23.7

To confirm that the six second trimester samples from fetuses with neural tube defects did not have dramatically different non-coding transcriptomes from the other second trimester samples, multidimensional scaling using Principal Component Analysis was performed on their expression of placentally expressed sncRNAs. When plotted for the first two principal components, the samples with neural tube defects were not distinct from the other second trimester samples (Fig. 3c). The possibility that some preterm samples would have developed observable neural tube defects or other placental or fetal dysfunctions had the pregnancies progressed further cannot be excluded. However, the lack of outliers in the first two principal components indicates, based on the non-coding transcriptomic data that we present, that none of the samples were significantly altered at the time of sampling (Fig. 3c).

## Data Availability

All data for this project were processed in MATLAB R2017b. The code used to process these data has been deposited in the Figshare repository, and is publicly available^28^.

## Online-only Table

**Online-only Table 1:** Sequences and genomic locations of novel placentally expressed miRNAs.

Sequence	Chromosome	Start*	Stop*	Mean RPM	MiRMaster-Assigned Probability	NovoMiRank Score
AACGUGGCCAAUGUGAUCUGGA	17	59,677,126	59,677,178	0.60	0.764	0.771
AACUGGGCAUGUUGGAACUAAGC	4	7,085,387	7,085,450	0.97	0.736	0.865
AAGAUUCUCUGAAGGUGUAGAGC	X	150,811,277	150,811,340	0.44	0.716	0.773
AAGUUUCUCUGAAAGUGUAGAGU	3	32,694,174	32,694,237	34.89	0.705	0.965
AAGUUUCUCUGAACGUGUAAAG	10	87,050,250	87,050,311	2.94	0.696	0.795
AAGUUUCUCUGAACGUGUAGAGC	17	19,190,179	19,190,240	83.76	0.807	0.751
AAGUUUCUCUGAAUGUGUAGAA	3	98,872,116	98,872,176	10.08	0.708	1.015
ACAGAAAUCUUGAUCAAUAACC	17	59,274,104	59,274,167	1.59	0.722	1.203
ACCAAAGGAGCUCUGAGAGAGG	2	46,825,201	46,825,276	0.76	0.681	1.245
ACCCAGGAGGUGGAGGUUGCAGU	2	86,123,183	86,123,241	1.05	0.717	1.013
ACCGGAGGCAGAGGUUGCAGU	22	20,025,129	20,025,188	0.70	0.732	1.001
ACUAAGCACCUGAAGUCAGAA	12	122,421,023	122,421,095	1.13	0.749	1.151
ACUCUACAGUGGACAGCUUUU	9	32,456,307	32,456,370	2.12	0.760	1.064
AGACCUACUUAUCUACCAACAG	15	82,756,010	82,756,069	17.47	0.675	0.997
AGGUAGAUAGAACAGGUCUUG	15	82,756,010	82,756,069	128.05	0.675	0.997
AGGUUGGAGUGUGGGAACUGUU	22	41,336,166	41,336,220	0.61	0.652	1.025
AGUUUCUCUGAAAGUGUAGAGC	4	159,507,392	159,507,453	0.43	0.783	0.762
AUAUGCUGGGCACUGUUCAAGAA	2	36,658,127	36,658,205	0.54	0.708	0.967
AUCGAGGCUAGAGUCACGCUUGG	11	8,684,227	8,684,288	1.38	0.774	0.847
CAAAAACUGUGAUUACUUUUGC	4	154,218,922	154,218,980	1.86	0.677	0.860
CACUCCAGCCUGGAUGACAG	14	54,409,812	54,409,872	1.47	0.660	0.959
CAGUACAGAUCCUCGAGGUGUG	20	40,688,554	40,688,612	1.02	0.700	0.886
CCACGGUGGACUUAGCUCUGA	11	73,235,751	73,235,810	1.35	0.714	0.823
CCACUGCACUCCAACCUGGGUG	6	1,190,049	1,190,107	0.97	0.847	0.794
CUACGUGACCAGAAGUUUCAG	4	128,550,153	128,550,214	0.60	0.755	0.853
CUCAGAGUUCCACCAGAUCAAU	21	40,563,376	40,563,435	0.35	0.817	0.773
CUGGCAUACCAGAUUUCUGGG	12	122,695,928	122,695,985	2.24	0.740	0.779
CUUGCUUGGGACUCUGGACGCA	15	49,155,876	49,155,932	0.55	0.656	0.826
GAUCAGGAGUUCAAGACCAGCC	13	59,106,128	59,106,189	3.44	0.801	0.945
GCAAAAACCGCAGUUACGUUU	3	76,343,543	76,343,596	0.97	0.699	0.951
GCAGUCCCAUUAGAUGCUGGCG	6	158,772,370	158,772,434	1.78	0.789	0.706
GCUCUACACUUUCAGAGAAACUU	4	159,507,392	159,507,457	409.16	0.803	0.749
UCAAUGUGCGUGAGAAGUCAAAGC	17	19,283,438	19,283,504	3.38	0.678	1.140
UCACCUGAGGUCCCCAUCUGA	15	74,199,665	74,199,730	0.55	0.677	1.274
UCAGGAUCAGGAUCUGCAGAGU	21	32,730,987	32,731,044	1.17	0.818	0.724
UCCCUAGUCUACUGGAGGAUAA	8	98,393,667	98,393,729	0.72	0.822	0.999
UCCGUCUGUUGCCCAGACUUCA	10	95,720,252	95,720,313	1.24	0.677	1.077
UCUGCUGAGAGUUUCUGACUGA	3	134,066,890	134,066,950	0.56	0.858	0.675
UCUGGUUGCUGUGUUGCAGAAA	17	17,818,264	17,818,329	5.78	0.678	1.020
UCUGUUUAGCAUCCUACAACCU	3	143,783,125	143,783,184	0.91	0.833	0.935
UGAUCUGGUGGAACUCUGAGAA	21	40,563,376	40,563,435	0.51	0.817	0.773
UGUUGUCGUUCCCCCUGCUUAA	3	186,787,299	186,787,359	0.72	0.660	0.778
UUAUGCCUGUGACUGCUGUAG	8	15,545,840	15,545,898	2.80	0.761	0.933
UUAUUGAUCAAGAUUUCUGUAG	17	59,274,104	59,274,167	0.53	0.722	1.203
UUCCAGUCCUUCUCGGAUGAAG	7	157,410,913	157,410,971	1.33	0.694	1.012
UUCUCAGGAUUCACACUUGUAG	19	51,682,100	51,682,160	0.93	0.889	0.799
UUCUGCAAAGCACACUCUCAGA	3	111,213,594	111,213,662	0.36	0.742	1.089
UUUUGCACAAAGAGUCUCAUGU	2	188,974,455	188,974,511	0.45	0.822	0.777

*The values in these columns indicate the positions of the first and last nucleotide of each sequence’s miRNA precursor.
